# Influence of Ethanol as a Co-Solvent in Cyclodextrin Inclusion Complexation: A Molecular Dynamics Study

**DOI:** 10.3797/scipharm.1412-08

**Published:** 2015-02-09

**Authors:** Kanokthip Boonyarattanakalin, Helmut Viernstein, Peter Wolschann, Luckhana Lawtrakul

**Affiliations:** 1 School of Bio-Chemical Engineering and Technology, Sirindhorn International Institute of Technology, Thammasat University, Pathum Thani 12121, Thailand; 2 Department of Pharmaceutical Technology and Biopharmaceutics, Faculty of Life Sciences, University of Vienna, Althanstrasse 14, 1090 Vienna, Austria

**Keywords:** Cyclodextrin, Inclusion Complexation, Co-solvent, Molecular Dynamics Simulation

## Abstract

Molecular dynamics (MD) simulations were used to investigate the dynamics and host-guest interactions of the inclusion complexes between a potent anti-HIV agent, UC781, and three different types of cyclodextrins (CDs) including βCD, 2,6-dimethyl-βCD (MβCD), and 2-hydroxypropyl-βCD (HPβCD) in aqueous solution with ethanol (EtOH) as a co-solvent. The MD simulation results revealed that EtOH as the co-solvent and the type of cyclodextrin affected the inclusion complex formation. From this study, UC781/MβCD provided the most stable inclusion complex. The competition for the cavity of βCD between UC781 and EtOH and the ensuing occupation of βCD cavities by EtOH resulted in a weaker interaction between βCD and UC781. In HPβCD, a supramolecular complex of UC781−HPβCD−EtOH was formed. The EtOH could easily fill the residual void space of the interior of unoccupied HPβCD due to the movement of UC781. In MβCD, the strong hydrogen bond interactions between the UC781 amide group and the secondary hydroxyl groups of MβCD significantly stabilized the inclusion complex in the presence of EtOH.

## Introduction

*N*-[4-Chloro-3-[(3-methyl-2-buten-1-yl)oxy]phenyl]-2-methyl-3-furancarbothioamide (UC7 81) is a non-nucleoside reverse transcriptase inhibitor (NNRTI) with potent inhibition of HIV-1 replication in cell culture systems (50% effective concentration (EC50) only about 3.0 ng/mL) [1–4]. UC781 has been tested in animals and in Phase I clinical trials in humans [5–7]. Therefore, this compound potentially can be used for the treatment and prevention of HIV-1 infection. However, the molecule has poor aqueous solubility of only 0.2 mg/L [8].

A well-established practice to increase aqueous solubility of poorly soluble drugs is the inclusion complexation by various cyclodextrins (CDs) [9–11]. These compounds have a relatively hydrophobic cavity and are, therefore, widely used as pharmaceutical excipients [12]. Various natural as well as chemically modified CDs are known with a broad variety of physico-chemical properties. Also, co-solvents play an important role in CD complexation as they influence the stability of the complexes and, moreover, they have a significant influence on the inclusion reaction mechanism [13, 14].

The improvement of the solubility of UC781 by inclusion complex formation has been investigated experimentally [15]. UC781 can form inclusion complexes with CDs with a host:guest ratio of 1:1. CDs provide substantial increases in both aqueous solubility and biological activity of UC781. The utilization of inclusion complexes of UC781 with CDs potentially reduce the amount of UC781 required in a formulation of an active microbicide product without significant loss in biological activity.

Molecular dynamics (MD) simulations of the 1:1 inclusion complexes of UC781 in aqueous solution have already been performed by our group [16], as well as on the behaviour of β-cyclodextrin in various water-alcohol mixtures [17]. In the present study, simulations with three different types of CDs (β-cyclodextrin (βCD), 2,6-dimethyl-β-cyclodextrin (MβCD), and 2-hydroxypropyl-β-cyclodextrin (HPβCD)) in 10% and 50% (v/v) ethanol (EtOH) solutions were carried out in order to investigate the influence of EtOH as a co-solvent on the inclusion complex formation. The effect of the CD type on the inclusion complex formation was also inspected.

## Results and Discussion

**Fig. 1:**
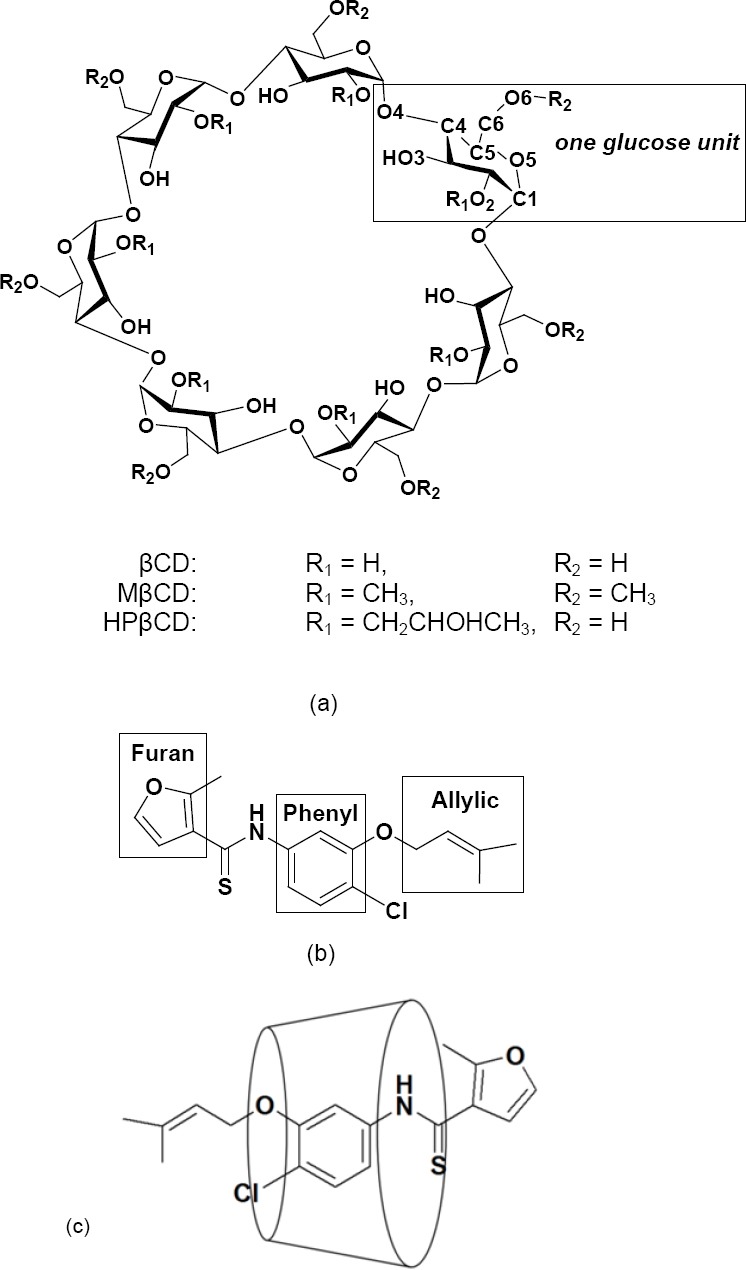
(a) Chemical structures and atomic numbering of βCD, MβCD, and HPβCD, (b) UC781, and (c) UC781/CDs inclusion complex.

**Fig. 2:**
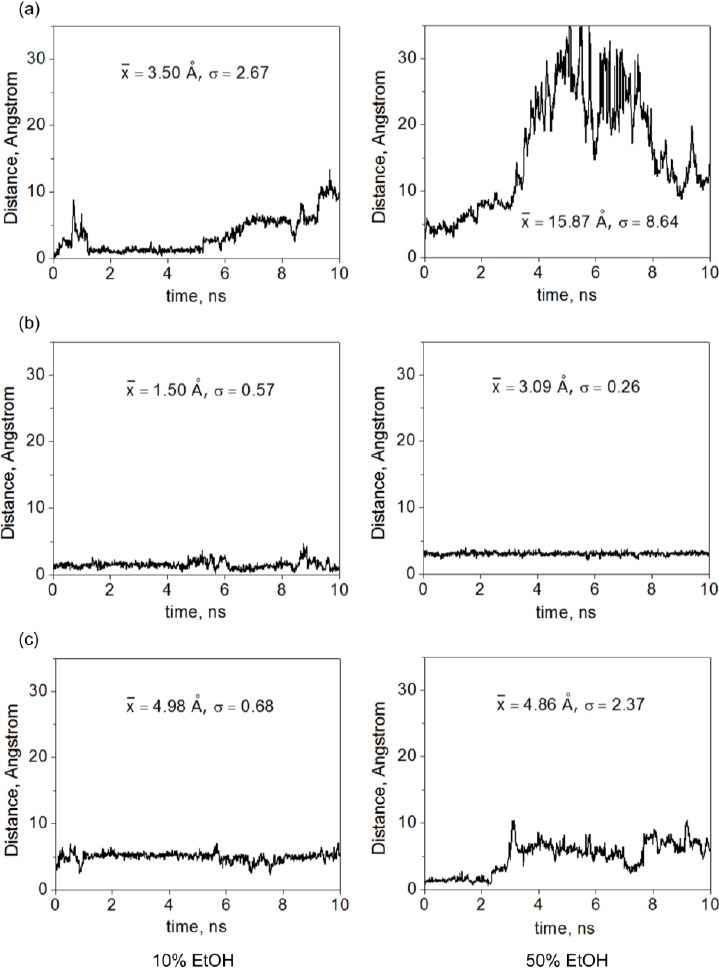
Distances between the centers of mass of UC781 and CD molecules of (a) UC781/βCD, (b) UC781/MβCD, and (c) UC781/HPβCD inclusion complexes in 10% and 50% ethanol solutions x̄ is the average distance and σ is the corresponding standard deviation.

The chemical structures of all CDs and UC781 are shown in Fig. 1. The motions of the UC781 molecule with respect to the CD cavity were observed by the measurement of the distance between UC781 and CD during the simulation time. These distances were determined from the centers of mass of UC781 to the center of mass of the CD ring. Fig. 2 shows the distances between UC781 and individual CD molecules at two concentrations of EtOH (10% and 50% (v/v)) as a function of the simulation time. Based on the molecular structure of UC781 in Fig. 1(b), the phenyl group can be considered as the center part of this drug molecule. The short distance between the center of mass of the guest and host molecules indicates the formation of a complex where the phenyl group stays inside the cavity of CD. Fig. 2 also gives some information about the movement of the drug molecule during 10 ns of simulation time. The optimized skeletal structure of βCD has the height of 6 Å which results in βCD’s center of mass at approximately 3 Å. Therefore, distances of less than 3 Å between UC781 and CD molecules were considered as inclusion complex formations in which the phenyl group of UC781 stayed inside the CD cavities.

At a distance larger than 3 Å and less than 6 Å, inclusion complexes can also be formed, but the phenyl ring of UC781 does not stay inside the CD cavities. The allylic or furan groups are inserted into the cavities of the CD molecules in this case.

For the UC781/βCD inclusion complex in EtOH (Fig. 2(a)), the distances between UC781 and βCD at a lower EtOH concentration show that an inclusion complex is possibly formed between 0 to 5 ns, then the value of the distance increases to approximately 6 Å to 10 Å which indicates a possible release of UC781 from the βCD cavity. However, at a higher EtOH concentration, a strong fluctuation of the position of UC781 can be observed. The distances are too far for the guest molecule to form a complex with βCD. This means that dissociation of the complex takes place even during this short simulation time.

The plots in Fig. 2(b) illustrate that an inclusion complex formation between UC781 and MβCD occurs at every time interval in both EtOH concentrations. However, at higher concentration, the distances between UC781 and MβCD slightly increase from the average of 1.50 Å at 10% to about 3.09 Å at 50% EtOH concentration, corresponding to standard deviations (σ) of 0.57 Å and 0.26 Å, respectively. The average distances show that at lower concentration, an inclusion complex is formed where the phenyl group remains inside MβCD’s cavity. At higher concentration, the phenyl group of UC781 slightly moves to the MβCD rim.

Fig. 2(c) describes the formation of inclusion complexes between UC781 and HPβCD. At a lower amount of EtOH, the distance between both molecules is about 4.98 Å with a SD around 0.68 Å. This distance value, together with the SD value, infer that the phenyl group of UC781 stays remote from the HPβCD center cavity with a certain distance, but does not dissociate from HPβCD. In a 50% EtOH concentration, the plot provides a short time period (0 to 3 ns) where the phenyl group of UC781 stays close to the center of the HPβCD cavity with a value of approximately 1.70 Å. After that, the guest molecule shifts to the HPβCD rim with an increasing value of the distance to approximately 6.17 Å.

The plots in Fig. 2 indicate that the UC781/MβCD inclusion complex is formed and it is stable over the entire 10 ns-simulation time at both concentrations of the co-solvent, while the UC781/βCD inclusion complex is not established at high concentrations of EtOH. Yang’s group [15] reported complexation constants (K_1:1_) of UC781 with different cyclodextrins in the ranking of MβCD>HPβCD>βCD (13449.7 M^−1^ for MβCD, 2239.4 M^−1^ for HPβCD, and 1119.5 M^−1^ for βCD). The value of K_1:1_ indicates the capability of the incorporation of UC781 into CD cavities forming 1:1 inclusion complexes. These results agree well with the simulations that UC781/MβCD inclusion complexes in both concentrations of EtOH can be formed and become stable for all 10 ns-simulation time periods as shown in the highest K_1:1_ of 13449.7 M^−1^. The K_1:1_ value of UC781/βCD is much lower than that of UC781/MβCD and UC781/HPβCD, which also agrees well with the simulations that the stable complex of UC781/βCD could be formed in the presence of EtOH.

**Fig. 3:**
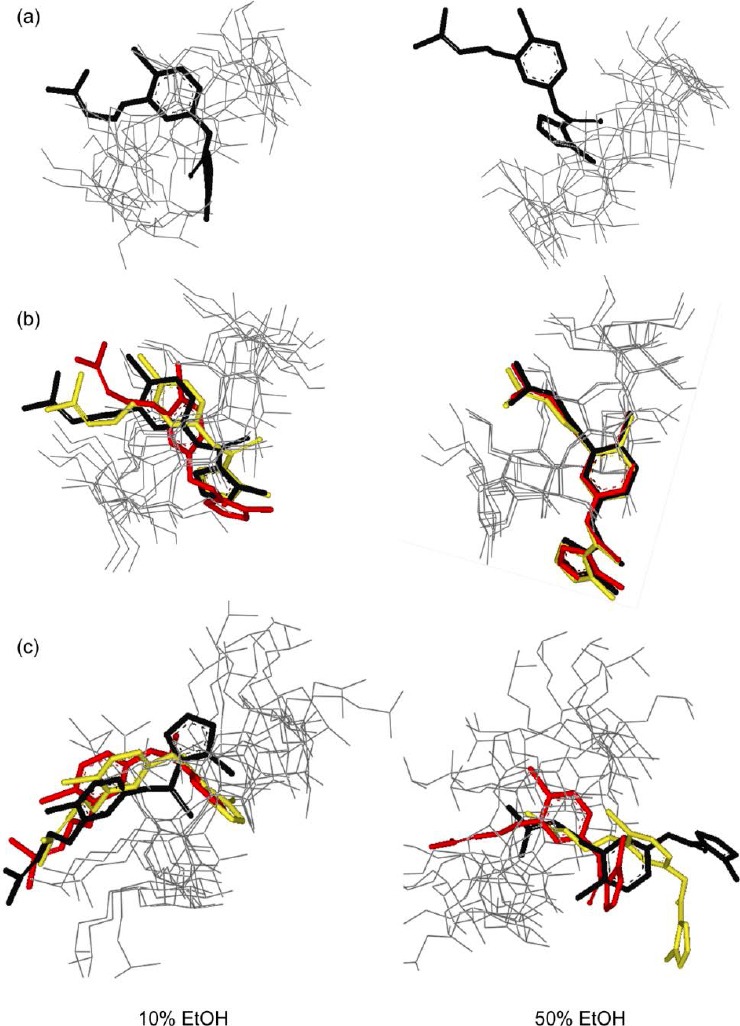
Representative structures of (a) UC781/βCD, (b) UC781/MβCD, and (c) UC781/HPβCD inclusion complexes in ethanol solutions. Black, light gray, and gray sticks represent the first, second, and third highest-populated clusters, respectively

**Fig. 4:**
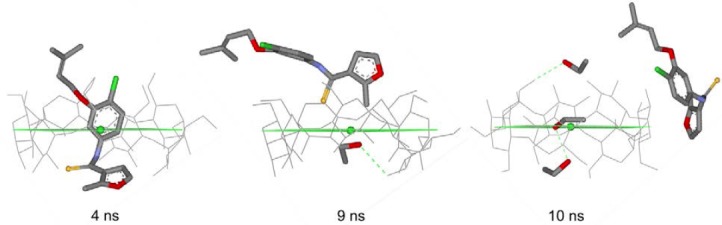
Conformation adjustment of the UC781/βCD inclusion complex in 10% EtOH solution (hydrogen atoms are omitted). The center of mass and the middle of the CDs are also indicated

Fig. 3 illustrates representative structures of the inclusion complexes UC781/βCD, UC781/MβCD, and UC781/HPβCD from the cluster analysis of molecular dynamics simulation snapshots at both concentrations of EtOH. Cluster analysis means that the snapshot geometries are sampled and ranked according to their populations. The structures of each inclusion complex were selected for Fig. 3 based on the highest population of possible configurations obtained from the molecular dynamics simulations. For the UC781/βCD inclusion complex, the highest populated configuration shows that the guest molecule stays inside the βCD cavity as shown in Fig. 3(a). However, at 50% EtOH, the location of the UC781 cannot be found at a high population inside the CDs’ cavity. This is in agreement with the result from Fig. 2, where large fluctuations of the distances between UC781 and βCD are observed. Fig. 4 illustrates the conformation adjustment of the UC781/βCD inclusion complex in 10% EtOH solution. In this trajectory, UC781 moves towards the narrow rim then leaves the βCD cavity, while EtOH molecules, which form hydrogen bonds with the secondary hydroxyl group at βCD wider rim (O2 and O3 – Fig. 1), move into the cavity. At high concentration of EtOH, the same incident as in the dilute solution occurs, but the guest molecule travels to the surrounding environment after 2 ns. The last snapshot structure of the UC781/βCD inclusion complex in Fig. 4 confirms that the addition of EtOH molecules inhibits UC781 complexation through the replacement of UC781 by EtOH. UC781 leaves the βCD cavity to be dissolved in EtOH-aqueous solution, while EtOH occupies the βCD cavity.

For the UC781/MβCD inclusion complex, the representative structures of UC781/MβCD inclusion complexes in both concentrations of EtOH are shown in Fig. 3(b). The presence of ethanol molecules in the solution seems to have no significant effect on the UC781/MβCD inclusion complex formation. Due to the weaker polarity of the surrounding solvent at higher concentration, UC781 moves slightly to MβCD’s wider rim in order to be at the suitable position that the formation of intermolecular hydrogen bonds between the amide nitrogen atom of UC781 and the secondary hydroxyl group of MβCD is enabled.

**Fig. 5:**
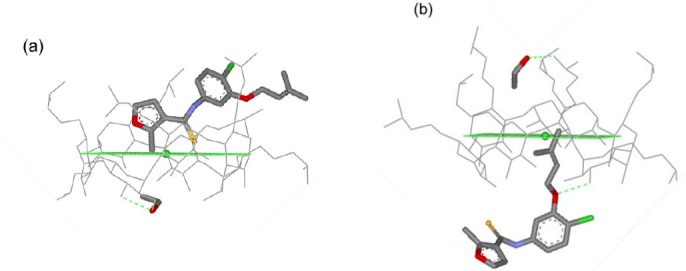
Supramolecular complexes of UC781−HPβCD−EtOH in (a) 10% and (b) 50% EtOH solutions (hydrogen atoms are omitted). The center of mass and the middle of CDs are also indicated

For the UC781/HPβCD inclusion complex, the different concentrations of EtOH affect the position of UC781 inside the HPβCD cavity (Fig. 3(c)). Fig. 5 shows that the contribution of EtOH upon UC781/HPβCD inclusion complexation depends on the EtOH concentration. In dilute solution, EtOH forms hydrogen bonds with the secondary OH groups at the HPβCD wider rim and moves into the cavity. UC781 moves to the narrow rim. In this case, the drug molecule can stay close to the narrow rim due to the intermolecular hydrogen bond between the phenyl oxygen of UC781 and the hydroxyl group at C6 position. Complex formation between HPβCD, UC781, and EtOH takes place. At higher concentration, the EtOH molecule, which forms hydrogen bonds to the hydroxyl groups of the hydroxypropyl substituent at HPβCD’s narrow rim, forces UC781 to shift to HPβCD’s wider rim to form a hydrogen bond there with the surrounding solvent molecules. In this case, the UC781/HPβCD inclusion complex incorporates EtOH at the HPβCD narrow rim to build a supramolecular complex.

Distortion of the shape of the CDs from a symmetric structure can be determined by the average distance between the O4(n) atoms of each glucose unit and the O4(n) mean plane which passes through all seven O4(n) atoms. Differences in O4(n) distances indicate the degree of distortion of the CD structure. The selected structural parameters corresponding to the standard deviations of CDs (βCD, MβCD, and HPβCD) in the inclusion complex are presented in Table 1. In the UC781/βCD inclusion complex, the value of the O4(n) deviation decreases when adding the co-solvent. As discussed above already, the presence of EtOH in the system inhibits inclusion complex formation. At higher concentration, the guest molecule detaches from βCD to dissolve in the EtOH-water mixture, then EtOH molecules replace the drug in the vacant βCD cavity. Therefore, it is implied that O4(n) deviations in the βCD structure come from the effect of inclusion complex formation.

**Tab. 1:**
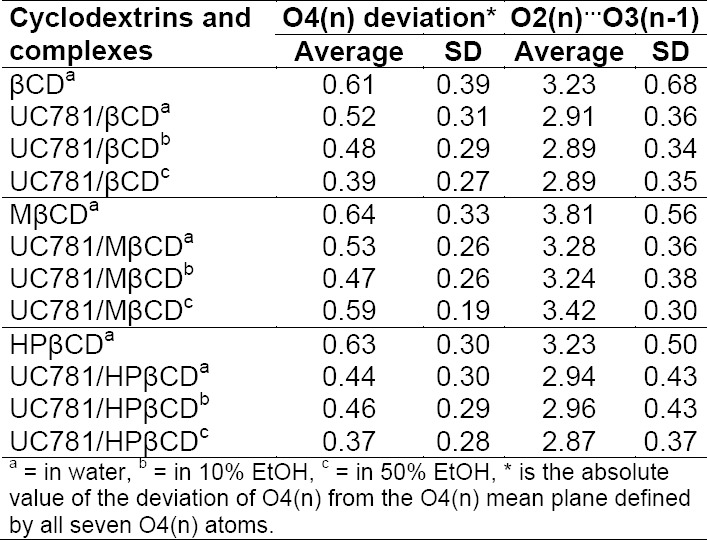
The distance (in Å) of free CDs and UC781/CD inclusion complexes in aqueous solutions

In the UC781/MβCD inclusion complex, the deviation of O4(n) decreases in the 10% EtOH concentration. However, at higher concentration (50% EtOH), the deviation increases. This result confirms that the formation of inclusion complexes with UC781 affects the deviation of the CD ring. The change in UC781 position with respect to the MβCD cavity results in the distortion of the MβCD molecule. At higher concentration, UC781 can move slightly to the MβCD wider rim due to the lower polarity of the surrounding solvent in order to stabilize the inclusion complex by the formation of intermolecular hydrogen bonds between the amide group of UC781 and the secondary hydroxyl group of MβCD.

In the UC781/HPβCD inclusion complex, the different position of UC781 with respect to the HPβCD cavity affects the deviations of HPβCD again. EtOH in the solution contributes to the location of UC781 in the HPβCD cavity. At dilute concentration, the position of UC781 locates closely to HPβCD’s narrow rim which gives a higher deviation of the HPβCD structure than the position where UC781 shifts to HPβCD’s wider rim at higher EtOH concentration (see Fig. 5).

The average distance between O2(n) and O3(n-1) of the three different CDs in the inclusion complex form are also reported in Table 1. The distances in the βCD and HPβCD complexes are shorter than those of the MβCD complex due to the flip-flop intramolecular hydrogen bond formation between the secondary OH groups at C2 and the adjacent C3 positions that are observed only in UC781/βCD and UC781/HPβCD throughout the simulations. None are observed in the case of the UC781/MβCD inclusion complex due to methylation at the C2 positions in MβCD.

## Conclusion

Molecular dynamics simulations of UC781/CD inclusion complexes in aqueous solution with EtOH as a co-solvent clearly show the effect of the co-solvent. Moreover, the influence of the type of cyclodextrin on inclusion complex formation can be differentiated. In βCD, the competition for the cavity of βCD between UC781 and EtOH and the ensuing occupation of βCD cavities by the alcohol molecules are the essential reason for the weaker interaction between βCD and UC781 in the presence of the co-solvent. In HPβCD, the supramolecular complex of UC781−HPβCD−EtOH occurs at both concentrations of the co-solvent. In MβCD, the strong hydrogen bond interactions between the amide group of UC781 and the secondary hydroxyl groups of MβCD significantly stabilize the inclusion complex in the presence of EtOH.

## Experimental

Initial molecular geometries of βCD and MβCD were obtained from the Cambridge crystallographic databases, ΒCDEXD10 [18] and CEQCUW [19]. The molecular geometry of UC781 was obtained from the Protein Data Bank, (1RT4, [20]). Hydrogen atoms were added to βCD, MβCD, and UC781 crystallographic structures by using the Accelrys DS Visualizer 2.0 program, and then optimized at the HF/6-31G(d,p) level, using the GAUSSIAN03 program [21]. For the HPβCD molecule, the starting structure was modified from the crystal structure of βCD by substituting all hydroxyl groups at C6 position with a 2-hydroxypropyl group. After that, the modified structure was fully optimized using the HF/6-31G(d,p) level.

The partial atomic charges of all molecules were derived using the restrained electrostatic potential (RESP) [22, 23] charge-fitting procedure. The ab initio electrostatic potential for RESP was calculated using the GAUSSIAN 03 program at the HF/6-31G(d) level of theory. Preparation of all force field parameters was done by using the Antechamber module of the AMBER program package. The AMBER 2003 force field [24] parameter sets were used for all the solute and solvent molecules.

The initial geometries of all inclusion complexes were obtained from the previous work of the MD simulations of UC781/CD inclusion complexes in aqueous solution [16]. Each UC781/CD inclusion complex was solvated by a truncated octahedron solvent box of a mixture of ethanol and TIP3P [25] water molecules. The numbers of solvent molecules and the size of the solvent box in both concentrations are presented in Table 2. Periodic boundary conditions were applied to the models in order to construct the matrix of repeating units for the system.

**Tab. 2:**
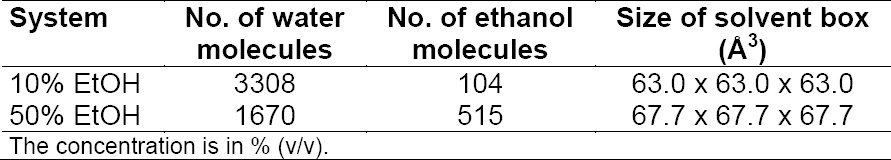
Number of solvent molecules

All MD simulations were performed using the AMBER10 program [26] with 2 fs time step size. The long range electrostatic interactions were treated by using the particle mesh of Ewald’s method [27] with a 10.0 Å cut-off distance. The SHAKE algorithm [28] was applied to constrain the bonds involving hydrogen atoms.

Energy minimizations of the models were performed using the steepest descent and then conjugate gradient algorithms. The solvated system was heated up from 0 to 300 K by gentle heating dynamics for 200 ps using restraints on the solute, followed by another 200 ps heating simulation on the whole system at 300 K with constant volume. After that, the Isobaric-Isothermal ensemble (NPT) was performed for 11 ns at the constant pressure of 1 atm and temperature of 300 K. The model was allowed to reach equilibrium for 1 ns, then the systematic coordinates were collected every 1 ps for another 10 ns of the simulation period. The LEaP, Sander, and Ptraj modules (in the AMBER program package) were used for preparing the input data, minimizing and MD simulating, and for analyzing the MD trajectories, respectively.

A total of 10,000 snapshots of the models in the simulations were used for further clustering analysis by the K-means cluster algorithm [29]. Clustering analysis was performed in order to obtain the representative structures for each UC781/CD inclusion complexe (UC781/βCD, UC781/MβCD, and UC781/HPβCD). The clustering trajectory frames were categorized into ten groups based on trajectory similarity considered by the root mean square deviation (RMSD), comparing the backbone atoms of CD and UC781. The highest populated clustering trajectory among the ten groups was chosen to be a representative structure of each UC781/CD inclusion complex in this study.
